# Comparison of miRNA expression patterns using total RNA extracted from matched samples of formalin-fixed paraffin-embedded (FFPE) cells and snap frozen cells

**DOI:** 10.1186/1472-6750-7-36

**Published:** 2007-06-29

**Authors:** Jinghuan Li, Paul Smyth, Richard Flavin, Susanne Cahill, Karen Denning, Sinead Aherne, Simone M Guenther, John J O'Leary, Orla Sheils

**Affiliations:** 1 Deptment of Histopathology, University of Dublin, Trinity College, Dublin, Ireland; 2 Applied Biosystems, Foster City, CA, USA

## Abstract

**Background:**

Archival formalin-fixed paraffin-embedded (FFPE) tissues have limited utility in applications involving analysis of gene expression due to mRNA degradation and modification during fixation and processing. This study analyzed 160 miRNAs in paired snap frozen and FFPE cells to investigate if miRNAs may be successfully detected in archival specimens.

**Results:**

Our results show that miRNA extracted from FFPE blocks was successfully amplified using Q-RT-PCR. The levels of expression of miRNA detected in total RNA extracted from FFPE were higher than that extracted from snap frozen cells when the quantity of total RNA was identical. This phenomenon is most likely explained by the fact that larger numbers of FFPE cells were required to generate equivalent quantities of total RNA than their snap frozen counterparts.

**Conclusion:**

We hypothesise that methylol cross-links between RNA and protein which occur during tissue processing inhibit the yield of total RNA. However, small RNA molecules appear to be less affected by this process and are recovered more easily in the extraction process. In general miRNAs demonstrated reliable expression levels in FFPE compared with snap frozen paired samples, suggesting these molecules might prove to be robust targets amenable to detection in archival material in the molecular pathology setting.

## Background

MicroRNAs (miRNAs) are small non-coding sequences of RNA, approximately 20 to 22 nucleotides long, which play important roles in the regulation of target genes by binding to complementary regions of messenger transcripts to repress their translation or regulate degradation [1]. This regulation appears to be involved in many fundamental cellular processes, including development, differentiation, proliferation, apoptosis, stress response, fat metabolism and insulin secretion [2]. Although the total number of different miRNA sequences in humans might approach 1000 based on the estimation of computer simulations [3], only 300 to 400 of them have been studied on fresh or snap-frozen samples to date. To discover the full regulatory impact of miRNA species and to understand individual biological functions within disease settings, larger scale analysis needs to be performed in a robust and reliable manner.

Formalin-Fixed, Paraffin-Embedded (FFPE) tissue samples are the most readily available archival material. They generally may be retrieved with documented clinico-pathological histories. Thus they represent an invaluable source for the study of human disease. However, these tissues have not been widely used in molecular biology due to the poor quality of RNA extracted from FFPE blocks [4] which is degraded to fewer than 300 bases in length [5] and also chemically modified by methylol groups during formalin fixation [6]. Thus, the value of FFPE materials in molecular setting has been shadowed by the technical difficulties limiting extensive analysis of gene expression. Interestingly, miRNAs are a class of small RNAs whose survivability and expression level in FFPE blocks compared with fresh tissues are largely unknown.

In this study we examined the reliability of miRNA detection in formalin fixed paraffin embedded blocks by interrogation of 160 miRNA assays in paired RNA extracts from fresh and FFPE samples using a cell line model.

## Results

### RNA extraction

To achieve 50 ng of total RNA for each RT reaction, 10,000 ng of total RNA (for 200 assays) was extracted from approximately 2 × 10^6 ^of FFPE cells and from approximately 1.7 × 10^5 ^snap frozen cells. Analysis using an Agilent 2100 Bioanalyser showed that the RNA Integrity Number (RIN Number) was 9.1 for the snap frozen cell preparations and 6.4 for the corresponding FFPE preparation.

### miRNA expression

**Figure 1:**
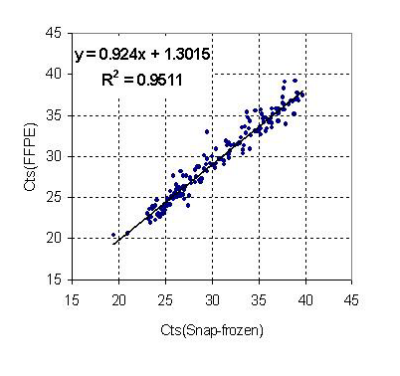
**Comparison of Ct values of 154 miRNA assays from paired FFPE and Snap-frozen cell lines**. Identical amount of total RNA was employed in each assay. R^2 ^is over 95% between two cell lines.

There was a good correlation of miRNA expression pattern in between FFPE and snap frozen cells, with R^2 ^> 0.95 (Figure 1). The mean of ΔCts was -1.04107. The median of ΔCts was -1.152, (126 below 0 and 28 above 0) with p < 0.0001. The sign test of median showed that miRNA exhibited approximately two fold higher expression with the total RNA extracted from the FFPE cells than that extracted from the snap frozen cells.

**Figure 2:**
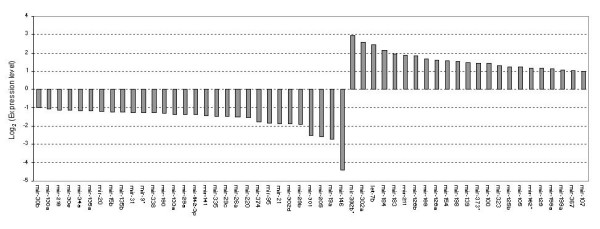
**Sorted Log_2_(Expression level) shows 30 miRNAs with decreased expression and 23 with increased expression in FFPE**. The most significantly altered expression was seen in mir-146 with decreased expression and mir-302b* with increased expression.

65.58% of ΔΔCts (101 out of 154 determined assays), were between +1 and -1. Furthermore the abundance of some individual miRNAs changed in FFPE cells with a total of 23 miRNAs displaying increased expression and 30 miRNAs decreased expression (Table 1 and Figure 2).

**Table 1:** Sorted expression levels of 160 miRNA between FFPE and snap frozen cells using ΔΔCts. 65.58% of ΔΔCts (101 out of 154 determined assays), were between +1 and -1

Decreased expression	Increased expression	ΔΔCts between +/-1				Undeter-mined
mir-30b	mir-302b*	mir-9	mir-133b	mir-200a	mir-370	c-lin-4
mir-130a	mir-302a	mir-10a	mir-134	mir-200b	mir-371	mir-104
mir-218	let-7b	mir-15a	mir-137	mir-200c	mir-372	mir-122a
mir-30e	mir-184	mir-17-3p	mir-138	mir-203	mir-373	mir-144
mir-34a	mir-183	mir-17-5p	mir-140	mir-204	let-7d	mir-302b
mir-135a	mir-211	mir-23a	mir-142-5p	mir-210	let-7e	mir-325
mir-20	mir-128b	mir-23b	mir-145	mir-213	mir-2	
mir-15b	mir-189	mir-25	mir-147	mir-214	let-7g	
mir-135b	mir-128a	mir-26b	mir-148a	mir-215	let-7i	
mir-31	mir-154	mir-27a	mir-149	mir-216	let-7a	
mir-9*	mir-198	mir-27b	mir-150	mir-219	mir-16	
mir-338	mir-139	mir-28	mir-151	mir-221		
mir-190	mir-373*	mir-30a-3p	mir-152	mir-222		
mir-133a	mir-100	mir-30c	mir-154*	mir-223		
mir-29a	mir-323	mir-30d	mir-155	mir-224		
mir-142-3p	mir-125b	mir-34b	mir-181a	mir-296		
mir-141	mir-105	mir-34c	mir-181b	mir-299		
mir-335	mir-182*	mir-92	mir-181c	mir-302c		
mir-29c	mir-129	mir-96	mir-182	mir-302c*		
mir-26a	mir-159a	mir-98	mir-185	mir-320		
mir-220	mir-199a	mir-99a	mir-186	mir-324-5p		
mir-374	mir-367	mir-103	mir-187	mir-326		
mir-95	mir-107	mir-106a	mir-191	mir-328		
mir-21		mir-124a	mir-193	mir-330		
mir-302d		mir-124b	mir-194	mir-331		
mir-29b		mir-125a	mir-195	mir-337		
mir-301		mir-126	mir-197	mir-339		
mir-205		mir-127	mir-199a*	mir-340		
mir-19a		mir-130b	mir-199b	mir-342		
mir-146		mir-132	mir-199-s	mir-368		

## Discussion

Since their discovery, miRNA analysis has generally been performed on snap-frozen or fresh samples, using variable techniques including microarray, northern blot analysis and PCR [2]. FFPE tissues, as a readily available source, could be invaluable in performing miRNA expression analysis if their expression were maintained following processing. In this study we compared miRNA profiling performed on fresh samples and FFPE using stem-loop RT-PCR quantification techniques in a cell line model. We found that miRNA profiling could be performed on routinely fixed FFPE blocks.

### miRNA abundance in FFPE

Some laboratories have examined mRNA gene expression profiles using real-time RT-PCR in paired snap-frozen and FFPE tissue samples [7-10]. The general consensus is that mRNA detection from archival material is limited due to the labile nature of mRNA and the deleterious effects of enzymatic fragmentation during long periods of storage and RNA modification induced by formalin fixation. Subsequently, it has been suggested that small amplicons [11] (shorter than ~ 130 [7,9] nucleotides) could have utility as robust markers in gene expression studies using FFPE tissues. Indeed, our own experiments (data not shown) confirmed this phenomenon using mRNA targets over a range of amplicon sizes in this cell line model. For example, FFPE extracts produced Cts 4 to 10 cycles higher than their snap frozen counterparts depending on the amplicon sizes used (62 to 164 bp). Cts between FFPE and snap frozen were closer for small amplicons than that for large amplicons. For example analysis of GAPDH using a target amplicon of 67 bp displayed a mean difference of 4.28 cycles, whereas an assay designed for the same gene (GAPDH) using a target amplicon size of 122 bp displayed a mean difference of 6.51 Cts between FFPE and snap frozen material.

miRNAs have the advantage of small size, being only approximately 20 to 22 nucleotides long. In addition, they are protein protected by the RISC complex. Consequently they may not be as susceptible to RNA degradation as mRNA in FFPE tissues. Our results showed that the amount of miRNA in total RNA extracted from FFPE was greater than that extracted from snap frozen cells when the input amounts of total RNA were identical. The average quantity of miRNAs derived from total RNA extracted from FFPE was double (one Ct lower) that in snap frozen cells which is most likely a consequence of methylol cross-links between RNA and protein introduced during processing.

**Figure 3:**
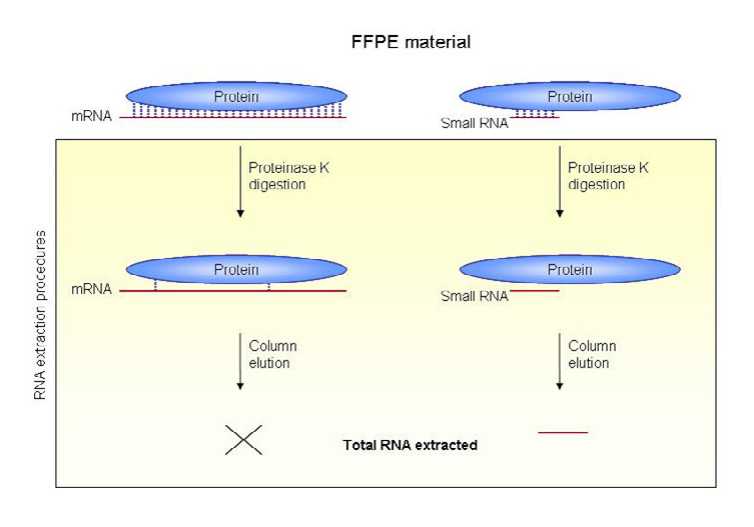
**A schematic representation of the impact of cross-links on RNA extraction**. In FFPE materials, RNA has been chemically modified by methylol groups to form cross-links with protein. Digestion with proteinase K [6] followed by column elution is the common method used to extract RNA from FFPE. However, a fraction of RNA remains impervious to extraction because of un-removed cross-links. The longer an RNA molecule is, the more likely cross-links will remain after the digestion procedure. Therefore, it is easier to extract small RNA molecules than larger ones from archival material.

We extracted identical quantities of total RNA (10,000 ng) for analysis. In practical terms this required input of almost ten times the number of FFPE cells (2 ×10^6 ^cells) compared to snap frozen (1.7 × 10^5 ^cells). This difference in extracted yields was consistent with previous reports. This suggests the amount of RNA that can be extracted from FFPE tissue represents only a fraction of that which is obtainable from fresh-frozen tissue [9]. The residual cross-links in every RNA molecule that have not been removed by proteinase K digestion prevent this RNA being extracted (Figure 3). The longer an RNA molecule is, the greater the likelihood that a cross-link still exists after the proteinase K digestion procedure. Therefore, small RNA molecules are more amenable to extraction than larger mRNA molecules resulting to a higher expression of miRNA in FFPE compared to that in snap frozen.

### Reliability of miRNA in FFPE

It is plausible to anticipate that miRNA species are less susceptible to RNA degradation associated with tissue processing than occurs with mRNA, and this formed the hypothesis to be tested in this study. We found a good correlation of miRNA expression levels between FFPE and snap frozen cells with R^2 ^> 0.95. Our data demonstrated that, for majority of miRNAs, the expression in FFPE is comparable with snap frozen cells. 65.58% of miRNAs displayed ΔΔCts in a range between +/-1 indicating that these normalized profiles were essentially identical between the two samples.

However, there was some outlying data where there was poor correlation between expression profiles for the paired snap-frozen and FFPE samples. The most significant of these was mir-146 with decreased expression and mir-302b* with increased expression. These changes could possibly occur during formalin fixation procedure or could also be caused by post fixation handling. mir-146 overexpression has been found in PTC tissues [12] and was also suggested to be involved in cellular stress [13] and innate immune responses [14]. Interestingly, we found it was decreased in FFPE extracted Nthy-ori cells. For those overexpressed miRNAs, it is possible that precursors of miRNA might have been cleaved by RNase to produce positive signals because FFPE blocks are often stored at room temperature in the absence of an RNase free environment. Alternatively, increased cellular stress following harvesting and during the fixation process may have contributed to the altered expression patterns in specific miRNAs. In these cases, the FFPE material could still be used to compare relative miRNA expression patterns if a series of known blocks were fixed and handled simultaneously or in the same manner.

## Conclusion

We analyzed 160 miRNAs expression levels in freshly fixed FFPE by comparing to snap frozen in a cell line model. Although the RNA extracted from FFPE blocks is often compromised, we demonstrated the robustness of miRNA profiles in FFPE material which could provide a source of study material for large scale or retrospective studies. This study has confirmed the proof of principle that miRNA species may be successfully extracted and analysed from archival sources. Further work may be required to determine precise effects of FFPE on miRNA expression profiles across different tissue samples.

## Methods

### Cell culture and formalin fixation

Nthy-ori 3-1 (ECACC, Wiltshire, UK) is a normal thyroid follicular epithelial cell line derived from adult thyroid tissue that has been transfected with a plasmid encoding for the SV40 large T gene [15].

This cell line was grown to confluence in a humidified atmosphere containing 5% CO_2 _at 37°C in the following plating medium: RPMI 1640 with 2 mM L-glutamine, 10% Foetal calf serum (FCS), Penicillin (100 U/ml) and Streptomycin (100 μg/ml). Tripsinized cells were counted with a hemocytometer. Suspended cells were aliquot and were pelleted (a) snap frozen and (b) formalin fixed and paraffin embedded into a cell block. When formalin fixation was required, a cohesive solid cell pellet was constructed using 20% agar. The cells were centrifuged in an eppendorf tube, and the supernatant was removed using a pipette. Approximately 30 μl of pre-warmed agar (60°C) was added to each tube. The solid cell pellet was formed within a few seconds. Cell blocks were fixed following standard tissue processing which included 10% buffered formalin fixation for 4 hours, on a Tissue-Tek^® ^V.I.P.™ tissue processor. The pellets were subsequently paraffin embedded.

### RNA extraction

RNA was extracted from fresh cells using mirVana™ miRNA Isolation kit (Ambion Ltd., Cambridgeshire, UK) and from FFPE cells using RecoverAll™ Total Nucleic Acid Isolation kit (Ambion Ltd., Cambridgeshire, UK) following the manufacturer's protocol. For snap-frozen extraction, one extraction was performed using approximately 4 × 10^5 ^cells. For FFPE extraction, 4 extractions were performed in parallel with one pellet (1 × 10^6 ^cells) in each extraction. The entire pellet was dissected from each block and was finely minced using a scalpel. These preparations were then deparaffinized, followed by proteinase K digestion for 3 hours at 50°C, on column DNase digestion and elution as described in the protocol. At that point all 4 FFPE extracts were combined into one tube designated the FFPE sample. The concentrations of these two samples were measured using a NanoDrop^® ^ND-1000 Spectrophotometer (Wilmington, USA) and extracts were diluted to 10 ng/μl. RNA quality was measured using the RNA 6000 Pico LabChip^® ^Kit on an Agilent 2100 Bioanalyser (Agilent technologies, Waldbronn, Germany).

### miRNA assays

Applied Biosystems TaqMan^® ^microRNA (miRNA) assays (designed for mature miRNA quantification using Applied Biosystems Real Time PCR instruments) were utilised in this study. The human panel early access kit (P/N: 4365381, Applied Biosystems) used in this study contained 160 individual assays and comprised two steps: Reverse Transcription (RT) and real time PCR. The stem-loop RT primer specifically hybridizes to a miRNA molecule and is reverse transcribed with a MultiScribe reverse transcriptase [16]. The RT products are then quantified using real-time TaqMan^® ^PCR.

Applied Biosystems High-Capacity cDNA Archive Kit (P/N: 4322171, Applied Biosystems, CA, USA) was used following manufacturer's protocol for reverse transcription. Each RT reaction contained 50 ng of extracted total RNA, 50 nM stem-looped RT primer, 1 × RT buffer, 0.25 mM each of dNTPs, 3.33 U/μl Multiscribe reverse transcriptase and 0.25 U/μl RNase Inhibitor. The 15 μl reactions were incubated in an Applied Biosystems Thermocycler in a 96-well plate for 30 min at 16°C, 30 min at 42°C, 5 min at 85°C and then held at 4°C.

For the Real-time PCR step, amplification was carried out using sequence specific primers on the Applied Biosystems 7900 HT Real-Time PCR system. The 20 μl reaction included 1.33 μl RT product, 1 × TaqMan^® ^Universal PCR Master Mix with no UNG (P/N: 4324018, Applied Biosystems) and 1 × TaqMan^® ^MicroRNA assays. The reactions were incubated in a 96-well optical plate at 95°C for 10 min, following by 40 cycles of 95°C for 15 s and 60°C for 1 min. The real-time PCRs for each miRNA were run in triplicate. hsa-let-7a was included as an endogenous control and cel-lin-4 was incorporated as a negative control.

### Statistical analysis

Replicates were omitted if Ct standard deviation was greater than 1.5. All 160 miRNAs were detectable with the exception of c-lin-4 in FFPE and c-lin-4, mir-104, mir-122a, mir-144, mir-302b and mir-325 in snap frozen sample. The data was collected using Microsoft Excel and was statistical analyzed using MINITAB^® ^14 on ΔCts with the formulas below:

ΔCt = Ct_Mean(FFPE) - Ct_Mean(Snap frozen)

ΔΔCt = ΔCt - ΔCt_Mean

Expression level = 2^-ΔΔCt^

## Authors' contributions

JL performed the RNA extraction and miRNA analysis and wrote original and final versions of the manuscript. PS, RF, SC helped with the miRNA analytical analysis of miRNA data and helped draft the manuscript. KD, SA carried out cell culture. SG helped with the analysis of the miRNA data. JOL and OS conceived the study and helped write the original and final versions of this manuscript. All authors read and approved the final manuscript.
